# Liposomes loaded with bioactive lipids enhance antibacterial innate immunity irrespective of drug resistance

**DOI:** 10.1038/srep45120

**Published:** 2017-03-27

**Authors:** Noemi Poerio, Francesca Bugli, Francesco Taus, Marilina B. Santucci, Carlo Rodolfo, Francesco Cecconi, Riccardo Torelli, Francesco Varone, Riccardo Inchingolo, Fabio Majo, Vincenzina Lucidi, Sabrina Mariotti, Roberto Nisini, Maurizio Sanguinetti, Maurizio Fraziano

**Affiliations:** 1 Department of Biology, University of Rome Tor Vergata Rome, Italy; 2 Institute of Microbiology, Fondazione Policlinico Universitario Gemelli - Catholic University of Sacred Heart, Rome, Italy; 3 Unit of Cell Stress and Survival, Danish Cancer Society Research Center, Copenhagen, Denmark; 4 Department of Pediatric Hematology and Oncology, IRCCS Bambino Gesù Children’s Hospital, Rome, Italy; 5 Department of Pulmonary Medicine, Fondazione Policlinico Universitario Gemelli - Catholic University of Sacred Heart, Rome, Italy; 6 Cystic Fibrosis Unit, Paediatric Hospital “Bambino Gesù”, Rome, Italy; 7 Department of Infectious, Parasitic and Immunomediated Diseases, Istituto Superiore di Sanità, Rome, Italy.

## Abstract

Phagocytosis is a key mechanism of innate immunity, and promotion of phagosome maturation may represent a therapeutic target to enhance antibacterial host response. Phagosome maturation is favored by the timely and coordinated intervention of lipids and may be altered in infections. Here we used apoptotic body-like liposomes (ABL) to selectively deliver bioactive lipids to innate cells, and then tested their function in models of pathogen-inhibited and host-impaired phagosome maturation. Stimulation of macrophages with ABLs carrying phosphatidic acid (PA), phosphatidylinositol 3-phosphate (PI3P) or PI5P increased intracellular killing of BCG, by inducing phagosome acidification and ROS generation. Moreover, ABLs carrying PA or PI5P enhanced ROS-mediated intracellular killing of *Pseudomonas aeruginosa*, in macrophages expressing a pharmacologically-inhibited or a naturally-mutated cystic fibrosis transmembrane conductance regulator. Finally, we show that bronchoalveolar lavage cells from patients with drug-resistant pulmonary infections increased significantly their capacity to kill *in vivo* acquired bacterial pathogens when *ex vivo* stimulated with PA- or PI5P-loaded ABLs. Altogether, these results provide the proof of concept of the efficacy of bioactive lipids delivered by ABL to enhance phagosome maturation dependent antimicrobial response, as an additional host-directed strategy aimed at the control of chronic, recurrent or drug-resistant infections.

Infectious diseases represent one of the main causes of morbidity and mortality worldwide, with developing countries presenting the highest rates of mortality, and developed high-income countries still struggling with providing adequate antimicrobial therapeutic options, due to the increasing emergence of antimicrobial drug-resistant bacterial pathogens^1^. In particular, the frequency and spectrum of antibiotic resistance in specific bacterial pathogens responsible for respiratory tract infections continues to increase worryingly, with particular concerns being focused on *Mycobacterium tuberculosis* and on Gram-positive (*Streptococcus pneumoniae, Staphylococcus aureus*) as well as Gram-negative bacteria (*Klebsiella pneumoniae, Enterobacter spp, Acinetobacter baumannii*, and *Pseudomonas aeruginosa*)^2^. Recently, a number of strategies targeting host immune response, rather than directly the pathogen, have opened-up novel possibilities of treatment, in particular for multidrug-resistant tuberculosis^1^. Examples of host-directed therapies under development are represented by cellular therapy using the patient’s own bone marrow-derived mesenchymal stromal cells, micronutrients and other immune-modulators, antimicrobial peptide inducers and checkpoint inhibitors, specific immune-based therapies, and therapeutic vaccines^1^.

Among the effector phases of innate immune response, phagocytosis represents the most important effector mechanism deputed to elimination of invading bacterial pathogens. The ingestion of bacteria is an alpha-actin dependent process starting with the invagination of the cell membrane to generate the phagosome, an intracellular vesicle enclosing the invading pathogen^3^. The phagosome is subjected to a series of fission and fusion events with other intracellular vesicles that cause its maturation to phagolysosome, the organelle ultimately deputed to microbicidal action^4^. The phagolysosomal maturation is driven by the topologically and temporally controlled expression of second lipid messengers, which can recruit signal proteins on the membrane of the maturing phagosome through recognition of specific lipid-binding domains^5^. Several second lipid messengers have been previously reported to promote phagolysosome maturation and intracellular mycobacterial killing^6^,^7^,^8^,^9^,^10^. Bacterial pathogens have also been reported to interfere with host lipid metabolism as a strategy to permit their intracellular survival^11^. For example, *Salmonella enterica* introduces inside host cells a phosphoinositide phosphatases, SigD, in order to degrade phosphatidylinositol 4,5 bisphosphate to phosphatidylinositol phosphate (PIP), thus interfering with phagosome maturation^12^. *Listeria monocytogenes*, through the regulation of PI3P levels, promotes the fusion of early endosomal compartments with the bacterial vacuole, before lysing the phagosome and escaping into cytoplasm^13^.

*Mycobacterium tuberculosis* (MTB), as well as the attenuated *M. bovis* Bacille Calmette-Guerin (BCG), interfere with phagolysosome maturation^14^,^15^ by depleting the phagosome of PI3P. Several mycobacterial products, including lipoarabinomannan (LAM), Secretory acid phosphatase (SapM), Zinc-dependent metalloprotease (ZMP), 6 kDa early secreted antigenic target (ESAT-6) and *Mycobacterium tuberculosis* Protein Tyrosine Phosphatase A (MptpA), act in concert^16^,^17^ to inhibit the conversion of the *Mycobacterium*-containing phagosome into a phagolysosome, where the bacteria would finally be degraded. Interestingly, the phosphatydilinositol analogue LAM inhibits the production of PI3P by Vps34 and the activation of Vps34 by calmodulin^18^, whereas SapM, and PtpA and PtpB, two phosphatases, hydrolyze PI3P to form PI^19^. Moreover, MTB has been reported to inhibit phospholipase D (PLD) activation and, in turn, production of phosphatidic acid (PA). This inhibitory activity was reported as strictly related to inhibition of phagosome maturation and intracellular mycobacterial survival^6^. These data suggest that a pharmacologic approach aimed at increasing the concentration of bioactive lipids on maturing phagosomes would counteract microbial strategies, and may ultimately increase intracellular pathogen killing. However, delivery of bioactive lipids within phagocytes is challenging, due to their pharmacokinetics characteristics. In this context, targeting macrophages for PA delivery has been described by using Janus-faced apoptotic body like liposomes (ABL)^20^. These liposomes were prepared in order to contain i) phosphatidylserine (PS) at the outer surface of the liposome membrane, to make them resembling apoptotic bodies, and ii) a bioactive lipid, such as PA, at the inner surface. By this strategy, it has been possible to rescue PA-dependent phagolysosome maturation and antimicrobial response to MTB, both *in vitro* and in a mouse model of TB *in vivo*^20^.

In the present study, we show that ABLs can be exploited to deliver selected second lipid messengers (i.e. PA, PI3P, PI5P, Arachidonic acid, sphingosine 1-phosphate, lysobisphosphatidic acid) known to positively regulate phagosome maturation^5^; their efficacy, as novel host-directed therapeutic approach, has been evaluated in models of i) bacterial interference of phagolysosome biogenesis, ii) genetically impaired phagolysosome-dependent antimicrobial response, and iii) drug-resistant pulmonary infections.

## Results

### ABLs carrying selected lipid messengers induce antimicrobial response in a model of pathogen- mediated interference of phagolysosome maturation

**Figure 1:**
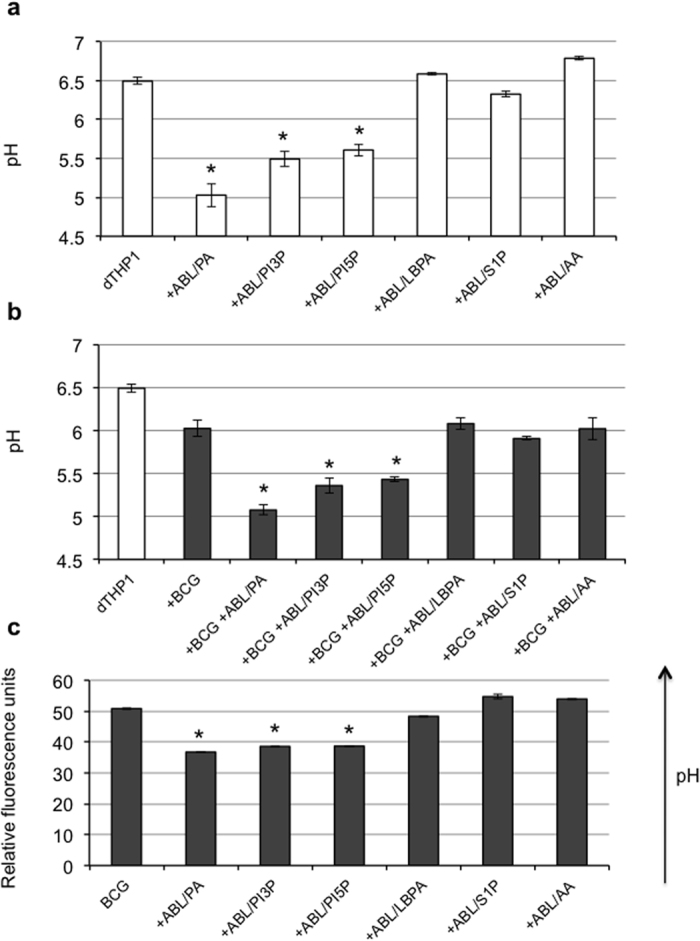
ABLs carrying selected bioactive lipids facilitate phagolysosome maturation. dTHP1 cells were uninfected (**a**) or infected (**b**) with BCG at the MOI of 5, treated with ABLs carrying the indicated bioactive lipid for 18 hours and then stained with Lysosensor green DND189 for pH determination. Results are shown as mean ± standard deviation of the values obtained from the triplicate of each condition and are representative of three separate experiments. *p < 0.001 in comparison with untreated control by one sided Student’s *t* Test. (**c**) BCG was labeled with 100 μg/ml of the pH sensitive dye NHS and used to infect dTHP1 cells (MOI 5). Infected cells were then treated with ABLs carrying the indicated bioactive lipid. The fluorescence expressed in relative fluorescence units is directly proportional to intraphagosomal pH. Results are shown as mean ± standard deviation of the values obtained from the triplicate of each condition and are representative of three separate experiments. *p < 0.001 in comparison with untreated control by one sided Student’s *t* test.

In the present study, the bioactive lipids PA, PI3P, PI5P, lysobisphosphatidic acid (LBPA), sphingosine 1-phosphate (S1P), or arachidonic acid (AA) chosen because of their capability to promote phagosome maturation^5^, were individually included in the inner layer of ABLs, to test their activity as enhancer of intracellular mycobacterial killing. The ABL preparations containing the selected lipids were preliminarily tested in flow cytometry for size distribution by comparing their forward scatter parameters (FS) with the FS of commercially available beads of known diameter. Results show a similar FS distribution for all ABLs tested (Supplementary Fig. 1d–i), which almost overlapped with the distribution of 1 μm beads (Supplementary Fig. 1c). Then, we analyzed the impact of the different ABLs on phagosome acidification of differentiated THP-1 (dTHP1) cells, infected or not with BCG. Results indicate that stimulation of dTHP1 with PA-, PI3P- or PI5P-loaded ABLs increases phagosome acidification to a pH of 5–5.5 in both uninfected (Fig. 1a) and BCG-infected (Fig. 1b) cells. This result was confirmed using NHS-labeled BCG. NHS is a pH sensitive fluorochrome whose fluorescence decreases proportionally to acidification of the microenvironment (Supplementary Fig. 2). Results show that when NHS–labeled BCG infected dTHP1 cells are treated with PA-, PI3P- or PI5P-loaded ABL, the mean fluorescence of BCG was reduced in comparison with untreated dTHP1 cells or with dTHP1 cells treated with other ABLs (Fig. 1c). As autophagy has been reported to be part of the innate antimicrobial armamentarium^21^, we assessed autophagy flux (the autophagy on-rate/off-rate) by monitoring LC3II cellular levels in the presence or absence of the lysosome inhibitor NH_4_Cl to allow LC3II accumulation^22^. Results show that intravacuolar acidification was associated with the enhancement of LC3II levels (Supplementary Fig. 3a,b), which occurs earlier (at 3 hours post-infection) in BCG-infected cells after stimulation with PI3P-loaded ABL, and later (at 18 hours post-infection) in uninfected cells after stimulation with PA-, PI3P- or PI5P-loaded ABL.

**Figure 2:**
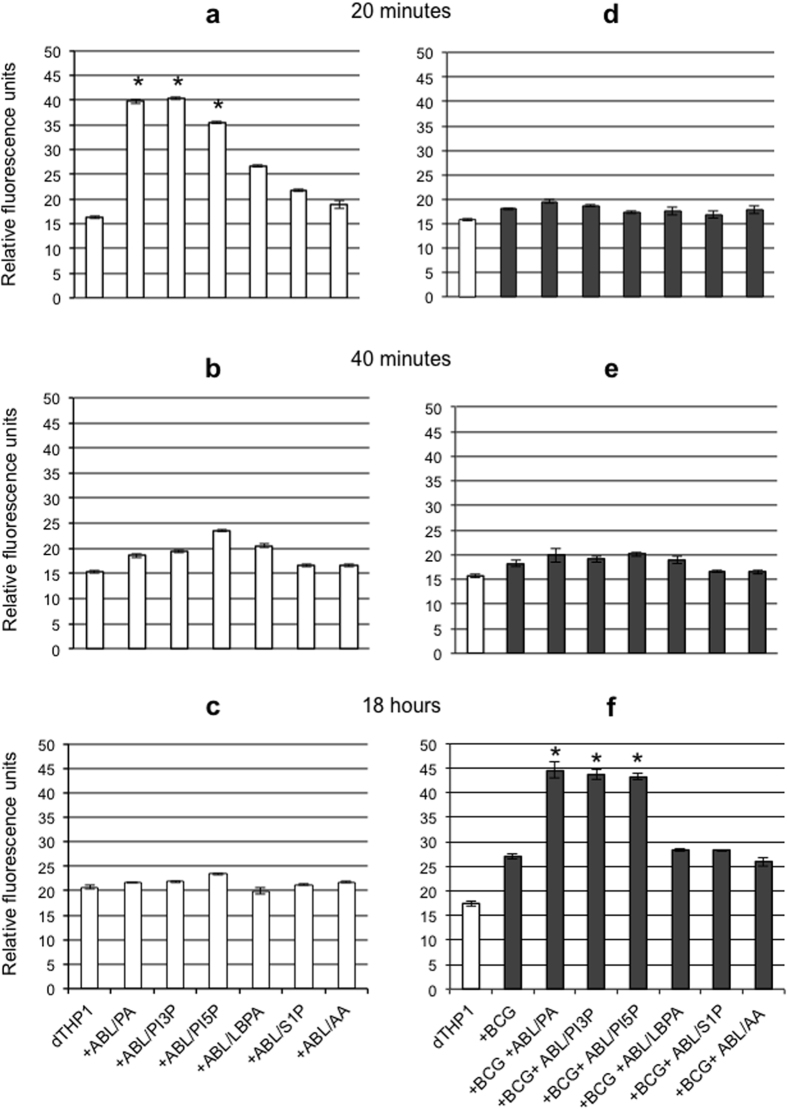
ABLs carrying selected bioactive lipids promote ROS production. Uninfected (**a–c**) or BCG infected (MOI 5) dTHP1 cells (**d**–**f**) were labeled with DCF at a concentration of 10 μM and subsequently treated with ABLs carrying the indicated bioactive lipid for 20 (**a**,**d**), 40 minutes (**b**,**e**) or 18 hours (**c**,**f**). The intensity of the fluorescence of DCF is proportional to the amount of ROS produced. The results are shown as mean ± standard deviation of relative fluorescence units performed in triplicates and are representative of 3 separate experiments. *p < 0.0001 in comparison with untreated control (**a**), or with BCG infected untreated control (**f**) by one sided Student’s *t* test.

Phagosome maturation and ROS generation are sequential steps leading to intracellular bacterial killing^23^. As type II NADPH oxidase (NOX-2) assemblies from component subunits on maturing phagosomes^24^, we monitored ROS generation following stimulation with the ABLs inducing phagosome maturation in both uninfected and BCG infected macrophages. In this context, PA-, PI3P- or PI5P-loaded ABLs were able to induce ROS generation in uninfected (Fig. 2a–c) and BCG infected macrophages (Fig. 2d–f), with a peak at 20 minutes (Fig. 2a) and 18 hours (Fig. 2f) after stimulation, respectively, suggesting the initial interference exerted by BCG with phagosome maturation and hence with NOX-2 assembly.

**Figure 3:**
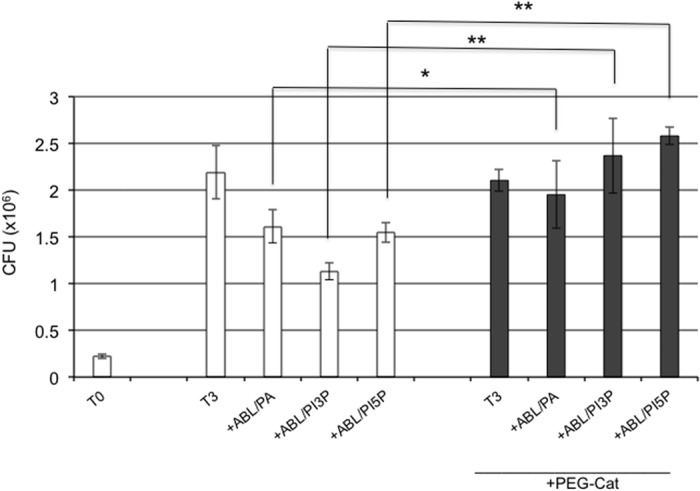
ABLs promote intracellular mycobacterial killing by a ROS dependent mechanism. dTHP1 cells were infected with BCG at the MOI of 5 and then treated for 3 days with ABLs carrying PA, PI3P, or PI5P in the presence or absence of 100 U/ml PEG-Cat. Results are expressed as mean ± standard deviation of CFU values performed in triplicate and are representative of two independent experiments. *p < 0.01 and **p < 0.0001 by one sided Student’s *t* test.

To measure the functional consequences of phagosome maturation and ROS generation, we measured BCG viability following stimulation with the same ABLs. In agreement with the above reported results, PA-, PI3P- or PI5P-loaded ABLs significantly enhanced intracellular mycobacterial killing, as detected by CFU assay (Supplementary Fig. 4a) or by BCG-lux assay in dTHP1 cells (Supplementary Fig. 4b) as well as in primary macrophages (Supplementary Fig. 4c). To confirm that the increased intracellular BCG killing induced by ABLs carrying PA, PI3P or PI5P was dependent by ROS generation, cells were exposed to pegylated catalase (PEG-Cat), which is known to convert hydrogen peroxide to water and oxygen, eventually reducing ROS activity. Results indicate that PEG-Cat almost completely abolishes the ABL-induced intracellular BCG killing (Fig. 3), highlighting that the main mechanism of ABLs action relies on ROS generation. Finally, the results obtained with PA-, PI3P- or PI5P-loaded ABLs were not associated to cytotoxic effects: viability of treated dTHP1 cells was almost unchanged if measured after 1, 3 and 5 days following ABLs stimulation and was indistinguishable from that of untreated cells (Supplementary Fig. 5).

### PA or PI5P delivered by ABLs rescue the impairment of antimicrobial immune response dependent upon CFTR inhibition

**Figure 4:**
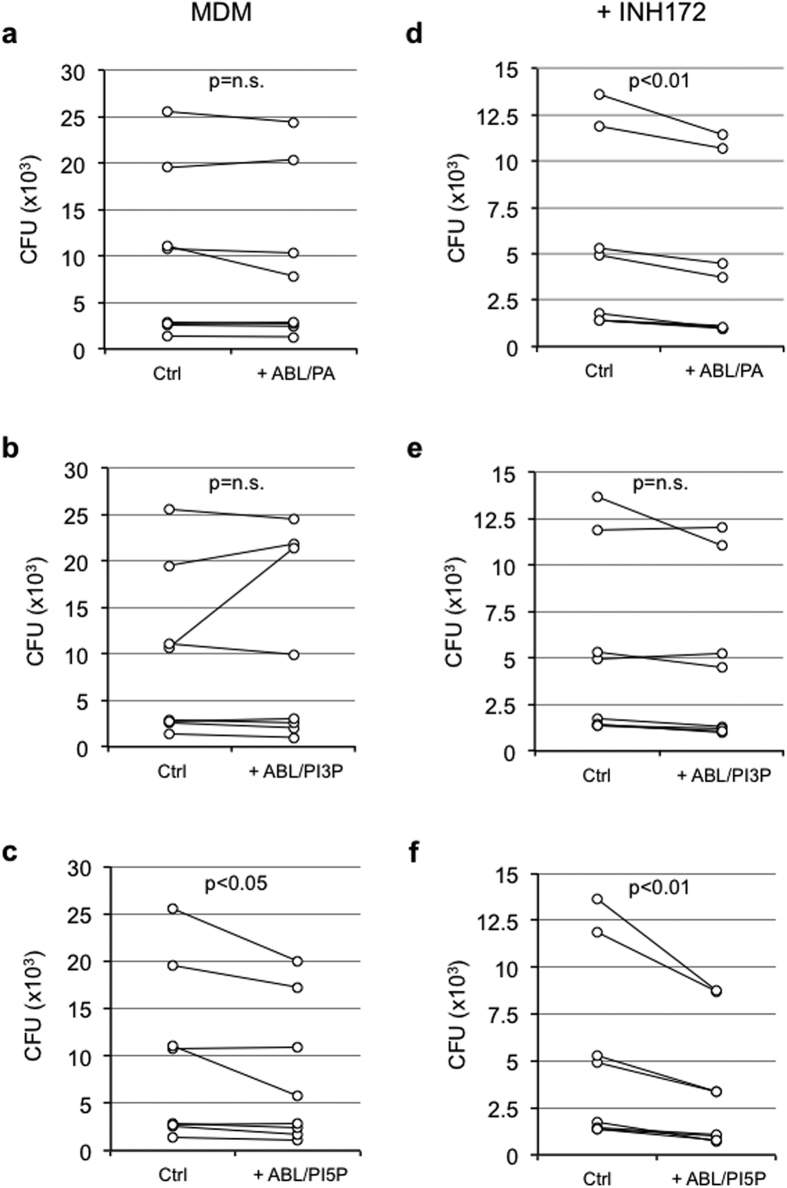
ABLs carrying PA or PI5P promote intracellular bacterial killing in primary macrophages expressing a pharmacologically inhibited CFTR. Monocyte derived macrophages (MDM) isolated from healthy subjects (n = 8) were infected with *P. aeruginosa*, as described in materials and methods, and then treated for 2 hours with the ABLs carrying PA, PI3P or PI5P in the absence (**a–c**) or in the presence (**d–f**) or of the CFTR inhibitor INH172. Bacterial growth was assessed by CFU assay. The results shown are obtained from triplicate for each condition. Statistical analysis was performed by using two sided Wilcoxon matched-pairs signed rank test and p value is indicated in the single panels.

**Figure 5:**
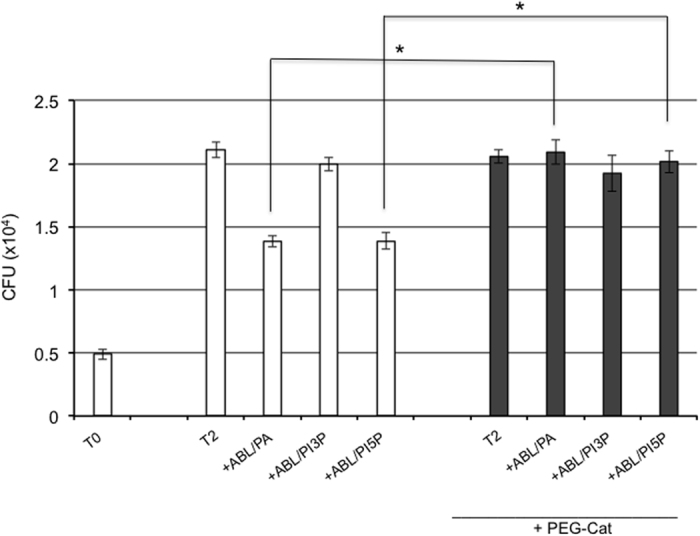
ABLs carrying PA or PI5P induce ROS dependent intracellular killing of *P. aeruginosa*. Primary monocyte derived macrophages (10^6^ cells/ml) were exposed to the CFTR inhibitor INH172 at a concentration of 10 μM and infected with *P. aeruginosa*, as described in materials and methods, and then treated for further 2 hours with the indicated ABLs in the presence or absence of catalase (PEG-Cat), used at the concentration of 100 U/ml. Bacterial growth was assessed by CFU assay. The results are shown as mean ± standard deviation of the values obtained from the triplicate of each condition and are representative of two separate experiments. *p < 0.0001 by one sided Student’s *t* test.

Cystic Fibrosis Transmembrane Regulator (CFTR) participates in phagolysosome biogenesis permitting intraphagosomal influx of chloride ions (Cl^−^) to balance the electric gradient that is generated by the vATPase-dependent proton accumulation in maturing phagosomes^25^. In the absence of Cl^−^ influx, the accumulation of positive charges prevents further acidification^26^ and macrophages show diminished antimicrobial response^27^. In this context, we preliminarily confirmed that the pharmacological inhibition of CFTR by exposure to INH172 affected the phagosome acidification of primary macrophages phagocytizing latex beads (Supplementary Fig. 6a) as well as their capability to kill the *P. aeruginosa* strain PAO1 (Supplementary Fig. 6b). Given their capacity to promote phagolysosome maturation and ROS-mediated bactericidal response, we tested ABLs carrying PA, PI3P or PI5P on primary macrophages from healthy subjects with pharmacologically inhibited or genetically mutated CFTR after *in vitro* infection with PAO1. Results show that the treatment of PAO1-infected primary macrophages with PI5P-loaded ABLs increased the intracellular bacterial killing capacity of macrophages with functional (Fig. 4c) or pharmacologically inhibited CFTR (Fig. 4f). This effect was specific for PI5P-loaded ABLs, since treatment with PA-loaded ABLs was only active on macrophages with pharmacologically inhibited CFTR (Fig. 4d), whereas PI3P-loaded ABLs were ineffective in this model (Fig. 4b,e). The mechanism of action of ABLs was confirmed to be dependent by the increased ROS production, since the treatment with ABL/PA or ABL/PI5P was ineffective in the presence of PEG-Cat (Fig. 5).

**Figure 6:**
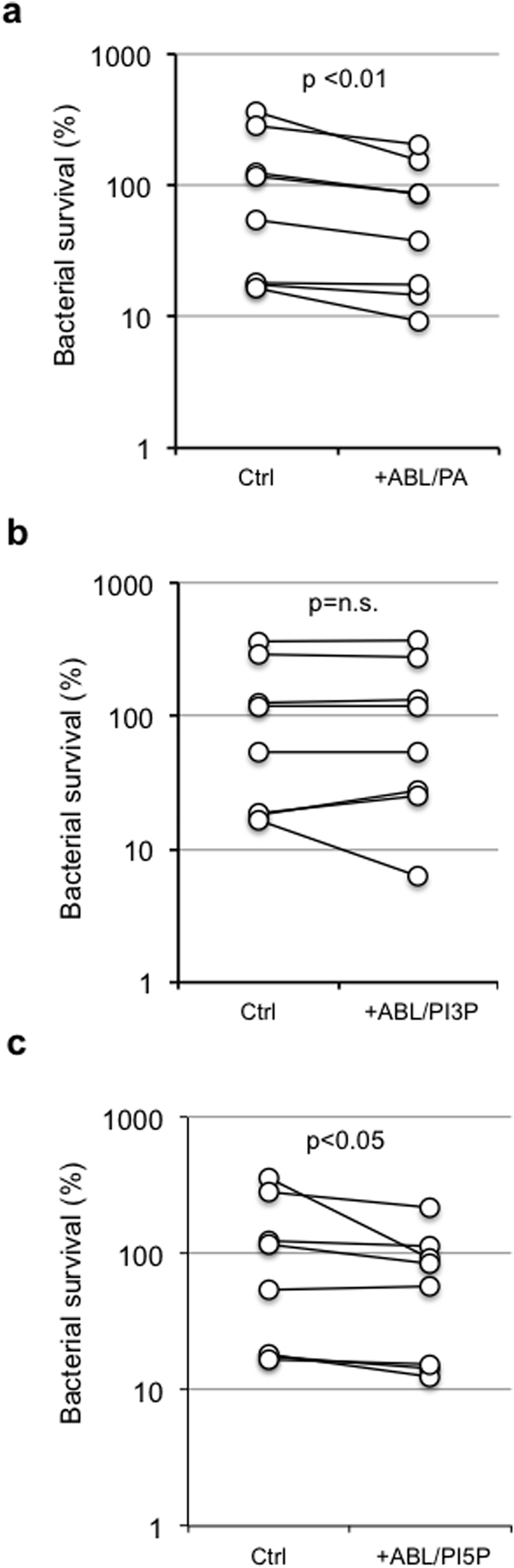
ABL carrying PA or PI5P promote intracellular bacterial killing in CF macrophages. Monocyte derived macrophages (10^6^ cells/ml), isolated from CF patients (n = 8), were infected with *P. aeruginosa*, as described in materials and methods, and then treated for further 2 hours with ABL/PA (**a**), ABL/PI3P (**b**), or ABL/PI5P (**c**). Bacterial growth was assessed by CFU assay. Survival is expressed as a percentage of bacterial survival and was calculated as the ratio between the CFU obtained after two hours of infection in the presence or absence of the indicated ABLs and the CFU obtained at time 0, before the addition of the ABLs. Statistical analysis was performed by using two sided Wilcoxon matched-pairs signed rank test and p value is indicated in the single panels.

The promising results obtained with PA- or PI5P-loaded ABLs on cells with pharmacologically inhibited CFTR prompted us to evaluate the consequences of ABL treatment on primary monocyte-derived macrophages isolated by 8 patients affected by cystic fibrosis (CF). Macrophages from CF patients were infected *in vitro* with PAO1, and bacterial killing was measured by CFU counting. Results show that ABLs carrying PA or PI5P (Fig. 6a,c) increase the killing capacity of macrophages with CF mutations, while the treatment with PI3P-loaded ABLs was ineffective (Fig. 6b), confirming data obtained in macrophages whose CFTR function was pharmacologically inhibited.

### ABLs carrying selected bioactive lipids enhance antimicrobial response in BAL cells isolated from patients with infectious pneumonia

**Figure 7:**
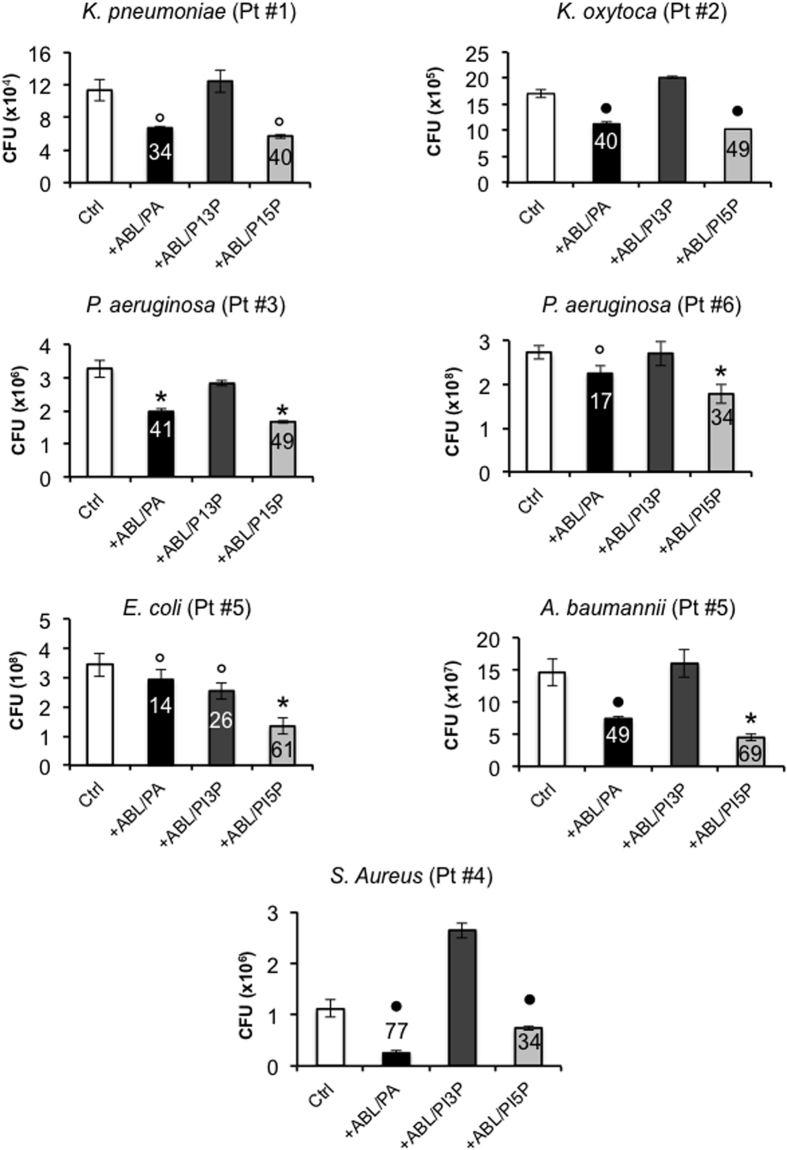
The microbicidal function of BAL cells from bacterial pneumonia patients is enhanced by ABLs. Bronchoalveolar lavage cells, from 6 patients with bacterial pneumonia, were treated for 18 hours with the indicated ABLs. Intracellular bacterial growth was evaluated by CFU. The number above each column indicates the percentage of killing is indicated above each column and was calculated by using the following formula: (1- CFU sample treated with ABLs/CFU untreated control) x100. The results are shown as mean ± standard deviation of the values obtained from the triplicate of each condition. ^○^p < 0.05, ^●^p < 0.001, and *p < 0.0001 in comparison with untreated cells by one sided Student’s *t* test.

The reported results suggest the feasibility of an ABL-mediated delivery of bioactive lipids into infected cells as a possible immunotherapeutic option to increase the innate cell-mediated clearance of pulmonary bacterial pathogens. As a preclinical model of efficacy, we therefore evaluated the efficacy of the ABL treatment in an *ex vivo* model of antibiotic resistant infections. The efficacy of ABLs carrying PA, PI5P or PI3P was tested in bronchoalveolar lavage (BAL) cells isolated from 6 patients affected by pneumonia, caused by the following different bacterial pathogens: *Klebsiella pneumoniae* (patient #1), *Klebsiella oxytoca* (patient #2), *Pseudomonas aeruginosa* (patients #3 and #6), *Staphylococcus aureus* (patient #4). Patient #5 had an *Escherichia coli* and *Acinetobacter baumannii* co-infection. Bacterial isolates had variable levels of antibiotic resistance (Tables 1 and 2) and *A. baumannii* was the most resistant, showing susceptibility to colistin, only (Table 1). Bacterial CFUs were measured after overnight culture of BAL cells treated or not with PA-, PI3P- or PI5P-loaded ABLs. Results show that a significant increase of intracellular killing of all bacterial pathogens was observed after treatment of BAL cells with PA- or PI5P-loaded ABLs, while treatment with PI3P-loaded ABLs was only effective in *Escherichia coli* infection (Fig. 7).

## Discussion

The emergence of drug-resistant bacterial pathogens with epidemic potential is a major global concern^1^. In 2015, an estimated 1.8 million people died worldwide from tuberculosis and 480000 new cases were reported with multi-drug resistant and extensively drug resistant tuberculosis^28^. Moreover, 23000 and 25000 deaths are attributable to antibiotic-resistant infections each year in the United States^29^ and in Europe^30^, and common etiologic agents are represented by multidrug- and pan-antibiotic- resistant Gram-negative and Gram-positive bacteria, such as *Pseudomonas* spp, *Acinetobacter* spp, *Streptococcus penumoniae, Staphylococcus aureus*, and Enterobacteriaceae, including endogenous bacteria. In one survey of US health centres, 78% of Gram-negative bacteria were resistant to all antibiotics except colistin (to which 62% of *Acinetobacter* spp, 59% of *Pseudomonas* spp, and 52% of *Enterobacter* spp were resistant)^31^. Many patients show increased susceptibility to these infections, including those who are immunosuppressed, receiving intensive care or with chronic lung pathologies such as bronchiectasis, chronic obstructive pulmonary disease and cystic fibrosis^32^. Notably, chronic respiratory infections are also the main cause of morbidity and mortality in patients with CF, which is the most common fatal single gene disorder. One of the hallmarks of these infections, led by the opportunistic pathogen *P. aeruginosa*, is their long-term (lifelong) persistence, despite intensive antimicrobial therapy, which reflects the rapid acquisition of multidrug resistance^33^. As a consequence, a growing awareness is emerging for the development of novel antibacterial agents and/or other immunotherapeutic options^2^.

In the present study, we have generated, tested and selected apoptotic body-like liposomes carrying bioactive lipids involved in phagosome maturation for their capacity to enhance antimicrobial response. The efficiency of these ABLs to improve bactericidal response was first tested in two models of inefficient phagosome maturation: the bacterial interference of phagolysosome biogenesis, i.e. the infection with BCG^34^,^35^, and the impaired phagolysosome dependent antimicrobial response, i.e. the model of CF^27^. In the model of BCG infection of macrophages, we showed that in addition to ABLs carrying PA, that were previously described^20^, also PI3P- or PI5P-loaded ABLs were able to significantly increase intraphagosomal acidification and to induce ROS production and ultimately promote intracellular mycobacterial killing in macrophages. PI3P has a well-known role in phagolysosome biogenesis and is critical for autophagy^21^,^36^. In fact, PI3P increase at the phagosome membrane level may recruit proteins associated with autophagy initiation, such as DCFP1 (double FYVE-containing protein 1) and WIPI (WD-repeat protein interacting with PI) proteins^37^. Moreover, PI3P appears to be important in the activation of NADPH oxidase in the phagosome, because it can interact with and activate the p40phox subunit of the enzyme^38^. PI5P has a not yet entirely defined mechanism of action, but it has been reported to be involved in different processes, such as (i) traffic regulation of intracellular vesicles and phagolysosome maturation through the stimulation of miotubularins^39^, (ii) chromatin-mediated control of gene expression^40^, (iii) cytoskeleton remodeling^41^ and (iv) activation of a non-canonical autophagy, which is independent of the class III phosphatidylinositol 3-kinase (PI3K), Vps34^42^. The mechanism of action of PA in phagolysosome maturation is also still unclear, but it appears to control membrane dynamics by directly affecting the physico-chemical properties of lipid bilayers independently of protein effectors^43^. In fact, PA is a cone shaped lipid, due to the small polar head in comparison to its hydrocarbon tail, and it can promote the membrane curvature and the subsequent fusion and fission processes, so favoring phagocytosis and phagosome maturation^20^,^44^ and the activation of NADPH oxidase^45^. Interestingly, acidification of phagosomes containing BCG was associated with LC3II accumulation after stimulation with PI3P-loaded ABL, only. These results support the crucial role played by PI3P in autophagy activation and in intracellular mycobacterial killing^35^ and suggest that, an autophagy-independent, phagolysosome maturation-dependent intracellular BCG killing is activated following stimulation with ABL carrying PA or PI5P.

The CF model was chosen as it is characterized by the impairment of mucosal immune response as a result of CFTR mutations^46^, whose malfunction is responsible for the block of phagolysosome maturation^25^ and for the reduced antimicrobial response^27^,^47^. We confirmed that the dysfunction of the CFTR channel caused by genetic mutation in CF patients or by pharmacological inhibition alters phagosomal acidification of macrophages and their microbicidal capability against a relevant CF related bacterial pathogen, such as *P. aeruginosa*. In this model, the use of PA- or PI5P-loaded ABLs restored the function of macrophages with pharmacologically inhibited CFTR or isolated by CF patients. The efficacy of PA- or PI5P-loaded ABLs has been also confirmed on BAL cells from patients with bacterial pneumonia, used as a clinically relevant model of drug resistant infection.

Together, these data are consistent with the key role of ROS in the intracellular killing of bacterial pathogens^24^. Although the mechanism through which PI5P may induce ROS generation is still not known and needs further investigation, the involvement of PA in the activation of NADPH oxidase in phagocytic cells has been reported^45^,^48^.In these last two models, we noticed a substantial inefficacy of the treatment of macrophages with PI3P-loaded ABLs in terms of intracellular bacterial killing. A possible explanation of this phenomenon is that PI3P conveyed by ABLs promotes autophagy, which contributes to protection against intracellular mycobacteria^35^, such as BCG. Other bacteria, such as, *P. aeruginosa* have been reported to trigger autophagy to escape intracellular killing^49^, and the induction of autophagy by PI3P delivered by ABLs may be irrelevant or even concur to this escape^50^. Similarly to *P. aeruginosa*, a strategy of autophagy manipulation has been described for *S. aureus* and *A. baumannii*, which reside in immature LC3II+ autophagosomes, as a niche for their replication^51^,^52^. Thus, bacterial pathogens may exploit different strategies to manipulate (auto)phagosome maturation and these features may probably reflect the differences observed with PA-, PI5P- or PI3P-loaded ABLs.

The ABL-enhanced antimicrobial activity in BAL cells represents a preclinical model of ABL efficacy, as the results are obtained in a cell microenvironment mirroring that of the infected lung and, hence, may be predictive of the possible *in vivo* action of ABL if delivered by aerosolic route^53^. The translational value of the results reported herein is further emphasized by the significant intracellular killing of different bacterial species characterized by a wide-spectrum of antibiotic resistance, such as *A. baumannii* of sample #5, for example, that showed resistance to all tested antibiotics except Colistin. Considering the global emergence of antibiotic resistance and the low number of new antimicrobial agents actually in clinical development, it is urgent to identify novel strategies aimed at simultaneously increasing the antimicrobial arsenal and preserving the already available drugs^54^. Together, the results reported herein represent the proof of concept of the feasibility of an effective and innovative host-directed therapeutic approach where PA or PI5P (or other selected bioactive lipids) may be delivered to effector phagocytic cells by aerosolized ABLs to enhance (auto)phagolysosome mediated antibacterial response. Although next *in vivo* validation in relevant animal models will be necessary and therapeutic regimens and dosage of administration need to be identified before of the transfer to clinical development, ABL based strategy may represent a promising therapeutic option which can be exploited to treat bacterial pathogens that have acquired or are expected to acquire antibiotic resistance.

## Methods

### Liposome preparation

ABLs were produced as previously described^20^. Briefly, the inner monolayer lipids (0.05 mg/ml) were suspended in anhydrous dodecane (Sigma). The following bioactive lipids were used for the inner monolayer: L-α-phosphatidic acid; 1,2-dioleoyl-*sn*-glycero-3-phospho-(1′-myo-inositol-3′-phosphate) (PI3P); 1,2-dioleoyl-*sn*-glycero-3-phospho-(1′-myo-inositol-5′-phosphate) (PI5P); D-*erythro*-sphingosine-1-phosphate (S1P); Lysobisphosphatidic acid (LBPA), (All by Avanti Polar Lipids) or Arachidonic Acid (AA) (Sigma). L-α-phosphatidylserine (PS) (Avanti Polar Lipids) was used as outer monolayer lipid and was added to a 99:1 dodecane:silicone solution to obtain a final concentration of 0.05 mg/ml. Asymmetric liposomes were prepared by adding 2 ml of outer monolayer lipid suspension over 3 ml of complete medium. Finally, 100 μl of the inner monolayer lipid suspensions were added over 2 ml lipid phase and the samples were centrifuged at 120 g for 10 minutes. After the centrifugation, ABLs were collected in an aqueous phase using a 5 ml syringe with a 16-gauge stainless steel needle. The following six different ABL formulations were produced: PS outside/PA inside (ABL/PA), PS outside/PI3P inside (ABL/PI3P), PS outside/PI5P inside (ABL/PI5P), PS outside/S1P inside (ABL/S1P), PS outside/LBPA inside (ABL/LBPA), and PS outside/AA inside (ABL/AA). Liposomes were then quantified by a flow cytometer FACSCalibur (Becton Dickinson), allowing quantification of monodispersed vesicles >0.2 μm in diameter.

### Cell culture

Human pro-monocytic THP-1 leukemia cell line was supplied by European Collection of Cell Culture, grown in RPMI 1640 containing fetal bovine serum (10%), gentamycin (5 μg/ml), L-glutammine (2 mM), nonessential amino acids (1 mM), sodium pyruvate (1 mM) and cultured in 75 cm^2^ polystyrene flasks. Before experiments, cells (5 × 10^5^ per well) were seeded in 24-well plates and cells were induced to differentiate by stimulation for 72 hours with Phorbol 12-Myristate 13-Acetate (PMA) (20 ng/ml), and used as a model of human macrophages.

Primary monocyte derived macrophages were prepared as previously described^47^. Briefly, peripheral blood mononuclear cells were isolated by healthy donors or Cystic Fibrosis patients and monocytes were separated, by using anti-CD14 monoclonal antibodies conjugated to magnetic microbeads (Miltenyi Biotec), according to manufacturer’s instructions. Monocytes were then suspended in complete medium and incubated for a further 5 days in 96-well plates at the concentration of 2 × 10^5^ cells/well in the presence of M-CSF (50 ng/mL) (R&D Systems) to get differentiated macrophages.

### Bacteria

*Mycoacterium bovis* BCG Pasteur strain (TMC1011) was grown and titred as described^14^. BCG transformed with the plasmid carrying luciferase gene (BCG-lux), kindly provided by Prof. R. Reljic from S. George’s University of London (UK), was grown as previously described^55^. *Pseudomonas aeruginosa* (PAO1 strain) were isolated by streaking on Pseudomonas isolation agar (PIA) (BD Difco TM), single bacterial colonies were collected and suspended in 15 ml of Luria-Bertani medium and grown in Erlenmeyer flask at 37 °C under stirring for 18 hours. The growth of bacterial cultures was monitored by measuring the optical density at the wavelength of 600 nm by a spectrophotometer (Ultrospec 1100pro, Amersham Biosciences). Bacilli were stored at −80 °C until use after suspension in Luria-Bertani medium and 30% glycerol.

### Infection and evaluation of intracellular (myco)bacterial growth

dTHP1 cells or primary macrophages were distributed in triplicate in 24 well plate (5 × 10^5^ cells per well) and exposed for 3 hours to BCG or to BCG-lux at the multiplicity of infection (MOI) of 5 or 10, respectively. After removal of extracellular bacilli, cells were treated with the indicated different ABLs added to a ratio of 1 to 1 (ABL:cell). Intracellular BCG growth was monitored by CFU assay^14^ or by luminometric assay^55^ performed after 3 hour exposure and at day 3 post-infection, by plating bacilli in triplicate after lysis of cells with saponin. Mycobacterial luminescence has been evaluated by Varioskan LUX Multimode Microplate Reader (Thermo Fisher Scientific).

Primary macrophages were distributed in triplicate in 96 well plates (2 × 10^5^ cells per well) and infected with *P. aeruginosa* for 1 hour at 37 °C at a MOI of 30 in the presence or absence of CFTR inhibitor (INH172, Sigma), used at the concentration of 10 μM. Thereafter, extracellular bacilli were killed by 1 hour incubation with gentamicin (400 μg/ml). Cells were then washed and incubated with PA-, PI3P- or PI5P-loaded ABLs, added to a ratio of 1 to 1 (ABL:cell), for further 2 hours in the presence or absence of INH172. Finally, cells were lysed with 1% deoxycholate (Sigma), samples diluted in PBS-tween 80 and CFU quantified by plating bacilli in triplicate on PIA. In order to evaluate the role of ROS in intracellular (myco)bacterial killing, BCG-infected cells or PAO1-infected cells were treated with PEG-Catalase (100 U/ml), as described^20^.

### Fluorometry

Phagosome acidification was assessed by using the fluorescent probe Lysosensor green DND 189 (Molecular Probes)^56^, which measures pH of acidic organelles such as phagolysosomes. Briefly, dTHP1 cells were infected or not with BCG and then treated or not with the different ABLs for 18 hours. Cells were then stained for 15 minutes at 37 °C with Lysosensor green DND 189 (1 μM). pH acidification was evaluated by fluorometry by setting the wavelength of excitation at 443 nm and emission at 505 nm according to the manufacturer’s instructions. Intraphagosomal acidification of vacuoles containing BCG was monitored by using BCG labeled with pH sensitive dye N-hydroxysuccin-imidyl 5-(and 6-)-carboxyfluorescein (NHS-CF) (100 μg/ml, Sigma), as described^57^. In particular, dTHP1 cells were infected in triplicate with NHS labeled BCG for 3 hours at the MOI of 5 and then treated overnight with the different ABLs. pH dependent fluorescence emission by NHS-labeled BCG was preliminarily confirmed by incubating BCG-NHS for 15 minutes at 37 °C in buffers calibrated at pH 4.5, 5.5, 6.5, 7.5 (from Intracellular pH Calibration Buffer Kit, Molecular Probes). The intensity of fluorescence was determined at an excitation wavelength of 492 nm and emission wavelength of 517 nm.

ROS generation was analyzed by loading cells with the fluorescent indicator 20,70-dichlorofluorescein diacetate (DCF) (Molecular Probes), used at the concentration of 10 μM, for 60 min at 37 °C in the dark. Thereafter, cells were infected or not with BCG, washed twice and treated with the different ABLs for 20, 40 minutes and 18 hours. The production of ROS was evaluated by fluorometry by setting the wavelength of excitation and emission at 488 nm and 530 nm, respectively.

Fluorescence has been evaluated by the use of a Varioskan LUX Multimode Microplate Reader (Thermo Fisher Scientific).

### Patients

Cystic fibrosis patients (n = 8) were enrolled at “Bambino Gesù” Children’s Hospital in Rome, Italy. All of the CF patients were clinically stable at the time of blood donation (5 ml). Controls (n = 8) were represented by buffy coats from healthy blood donors attending at the local Blood Transfusion Unit of Policlinico “Tor Vergata” in Rome. Clinical and demographic features of CF patients as well as healthy controls are summarized in Supplementary Table 1.

Patients with bacterial pneumonia (n = 6) were enrolled at the Department of Pulmonary Medicine of Fondazione Policlinico Universitario A. Gemelli, Rome. Clinical and demographic features of the patients are summarized in Supplementary Table 2. BALs were collected, following the guidelines of “American Thoracic Society Committee”^58^, at Bronchial Endoscopy Unit of Fondazione Policlinico Universitario Gemelli in Rome.

Bacterial isolates were identified by Matrix-Assisted Laser Desorption Ionization-Time-Of-Flight mass spectrometry (Bruker Corporation). Minimum inhibitory concentrations (MICs) were determined with the Vitek 2 (β-lactam inhibitor combinations, oxyimino-cephalosporins, carbapenems, aztreonam, quinolones, aminoglycosides), the E-test (bioMérieux, Inc) with cation-adjusted Mueller-Hinton agar (colistin), or Sensititre broth microdilution (Thermo Fisher Scientific) (tigecycline). Colistin MICs for *Enterobacteriaceae* isolates were classified using EUCAST breakpoints (susceptible, MIC ≤2 mg/L; resistant, MIC >2 mg/L)^59^. US Food and Drug Administration breakpoints were used for tigecycline MICs (susceptible ≤2 mg/L; resistant, ≥8 mg/L)^60^. Other MICs were interpreted according to Clinical and Laboratory Standards Institute breakpoints (http://www.eucast.org). Diagnostic quantitative culture thresholds were 10^4^ cfu/mL.

All patients gave written informed consent and the clinical studies were performed in accordance with the relevant guidelines and regulations and were approved by the local Ethics Committee. Cystic fibrosis patients, giving their (or parental) informed consent to participate to the study, were enrolled at “Bambino Gesù” Children’s Hospital in Rome after having received detailed information on the scope and objectives of the study by a sanitary personnel explained patient information leaflet (ethics approval #738/2014 of “Bambino Gesù” Children’s Hospital, Rome). Patients with bacterial pneumonia were enrolled at the Department of Pulmonary Medicine of Catholic University of Sacred Heart in Rome and were invited to participate through a patient information leaflet (ethics approval #20388/2013 of Catholic University of Sacred Heart, Rome).

### BAL cells and stimulation with ABLs

BAL cells were treated as previously described^20^ and suspended as 10^6 ^cells/mL in medium consisting of RPMI 1640 supplemented with 10% FBS, 2 mM L-Glu, 5 μg/ml Gentamycin, 5 μg/mL Ampicillin, and 2 μg/ml Fluconazole (all from Invitrogen) and incubated for 18 hours in 24-well plates in the presence or absence of the different ABLs used at the ratio of 1 liposome per cell. Respiratory bacterial specimens were quantitatively cultured on blood, chocolate, or MacConkey agars.

### Statistics

Comparison between groups was done using Student’s *t* test, as appropriate for normally distributed data. The Wilcoxon rank sum test was performed for data that were not normally distributed. Analysis of western blotting data has been performed by means of ANOVA and Bonferroni post-test to estimate the differences between selected samples. p < 0.05 was considered to be statistically significant.

## Additional Information

**How to cite this article:** Poerio, N. *et al*. Liposomes loaded with bioactive lipids enhance antibacterial innate immunity irrespective of drug resistance. *Sci. Rep.* **7**, 45120; doi: 10.1038/srep45120 (2017).

**Publisher's note:** Springer Nature remains neutral with regard to jurisdictional claims in published maps and institutional affiliations.

## Supplementary Material

Supplementary Information

## Figures and Tables

**Table 1:** Antibiogram of Gram- clinical isolates by BAL samples.

	*K. pneumoniae* (patient #1)	*K. oxytoca* (patient #2)	*P. aeruginosa* (patient #3)	*E. coli* (patient #5)	*A. baumannii* (patient #5)	*P. aeruginosa* (patient #6)
Ampicillin	≥32 (R)	16 (R)	≥32 (R)	≥32 (R)	≥32 (R)	≤2 (S)
Amoxycillin/Clavulanic acid	≥32 (R)	≤2 (S)	≥32 (R)	16 (R)	≥32 (R)	4 (S)
Piperacillin/Tazobactam	64 (R)	≤4 (S)	8 (S)	64 (R)	≥128 (R)	n.d.
Cefoxitin	≤4 (S)	≤4 (S)	n.d.	≤4 (S)	n.d.	n.d.
Cefotaxime	≤1 (S)	≤1 (S)	≥64 (R)	≥64 (R)	≥64 (R)	≤1 (S)
Ceftazidime	≤1 (S)	≤1 (S)	4 (S)	16 (R)	≥64 (R)	≤1 (S)
Cefepime	≤1 (S)	≤1 (S)	2 (S)	≥64 (R)	≥64 (R)	≤1 (S)
Ertapenem	≤0.5 (S)	≤0.5 (S)	≥8 (R)	≤0.5 (S)	≥8 (R)	≤0.5 (S)
Imipenem	≤0.25 (S)	≤0.25 (S)	1 (S)	≤0.25 (S)	≥16 (R)	1 (S)
Meropenem	≤0.25 (S)	≤0.25 (S)	2 (S)	≤0.25 (S)	n.d.	≤0.25 (S)
Amikacin	≤2 (S)	≤2 (S)	≤2 (S)	4 (S)	n.d.	4 (S)
Gentamicin	≤1 (S)	≤1 (S)	≤1 (S)	≥16 (R)	≥16 (R)	≤1 (S)
Ciprofloxacin	≤0.25 (S)	≤0.25 (S)	≤0.25 (S)	≥4 (R)	≥4 (R)	≤0.25 (S)
Tigecycline	1 (S)	≤0.5 (S)	n.d.	≤0.5 (S)	2 (i.e.)	n.d.
Fosfomycin	≤16 (S)	≤16 (S)	n.d.	≤16 (S)	≥256 (R)	n.d.
Nitrofurantoin	n.d.	n.d.	n.d.	≤16 (S)	n.d.	128 (R)
Colistin	≤0.5 (S)	≤0.5 (S)	≤0.5 (S)	≤0.5 (S)	≤0.5 (S)	1 (S)
Trimethoprim/Sulfamethoxazole	≥320 (R)	≤20 (S)	n.d.	≥320 (R)	≥32 (R)	≤20 (S)

(R) = resistant; (S) = sensitive; n.d. = not determined; i.e. = insufficient evidence that the organism or group is a good target for therapy with the agent.

**Table 2:** Antibiogram of *S. aureus* isolated by BAL sample (patient #4).

	*S. aureus* (patient #4)
Cefoxitin screening	positive
Benzil penicilline	≥0.5 (R)
Oxacilline	≥4 (R)
Gentamicin	≤0.5 (S)
Levofloxacin	≥8 (R)
Clindamicin inducible resistance	positive
Erithromicin	≥8 (R)
Clindamicin	0.25 (R)
Linezolid	2 (S)
Daptomicin	0.25 (S)
Teicoplanin	≤0.5 (S)
Vankomicin	1 (S)
Tetraclicin	≤1 (S)
Tigecycline	≤0.12 (S)
Fosfomycin	n.d.
Fusidic Acid	≤0.5 (S)
Mupirocine	n.d.
Rifampicin	≤0.03 (S)
Trimethoprim/Sulfamethoxazole	≤10 (S)
