# Supplementary material for: Liposomes loaded with bioactive lipids enhance antibacterial innate immunity irrespective of drug resistance
Source: Sci Rep. 2017 Mar 27;7:45120. doi: 10.1038/srep45120 (PMC5366871; doi:10.1038/srep45120)
Supplement: Supplementary Information [file srep45120-s1.pdf]

Supplementary Information

Liposomes loaded with bioactive lipids enhance antibacterial innate immunity irrespective of drug resistance

Noemi Poerio[1]†, Francesca Bugli[2]†, Francesco Taus[1], Marilina B. Santucci[1], Carlo Rodolfo[1], Francesco Cecconi[1,3,4], Riccardo Torelli[2], Francesco Varone[5], Riccardo Inchingolo[5], Fabio Majo[6], Vincenzina Lucidi[6], Sabrina Mariotti[7], Roberto Nisini[7], Maurizio Sanguinetti[2], & Maurizio Fraziano[1*]

[1]Department of Biology, University of Rome Tor Vergata;

[2]Institute of Microbiology, Fondazione Policlinico Universitario Gemelli - Catholic University of Sacred Heart, Rome;

[3]Unit of Cell Stress and Survival, Danish Cancer Society Research Center, 2100 Copenhagen, Denmark

[4]Department of Pediatric Hematology and Oncology, IRCCS Bambino Gesù Children's Hospital, Rome, Italy Department of Biology, University of Rome Tor Vergata, Rome, Italy.

[5]Department of Pulmonary Medicine, Fondazione Policlinico Universitario Gemelli - Catholic University of Sacred Heart, Rome;

[6]Cystic Fibrosis Unit, Paediatric Hospital "Bambino Gesù", Rome;

[7]Department of Infectious, Parasitic and Immunomediated Diseases, Istituto Superiore di Sanità, Rome;

†both authors contributed equally to the work.

Supplementary Methods

Dimensional analysis of ABLs by flow cytometry. The average diameter of ABL was assessed comparing the median forward scatter (FS) of produced ABLs with commercially available beads with the diameter of 0.6 and 0.8 μm (both by Sigma) and 1μm (Thermo Fisher), by the flow cytometer Gallios and analyzed by the Kaluza software (Beckman Coulter).

Cell viability assay. dTHP1 cells were stimulated with the different ABLs. At day 1, 3, and 5, cell viability was monitored by the MTT Cell Proliferation Assay Kit (Molecular Probe). The MTT assay is based on the cleavage of the yellow tetrazolium salt MTT (3-(4,5-Dimethylthiazol-2-yl)-2,5-diphenyltetrazolium bromide) to purple formazan crystal in metabolically active cells. The formazan is then solubilized, and the concentration determined by optical density at 540 nm. The assay is sensitive with the colorimetric signal proportional to the viable cell number.

Western Blotting. Whole cell extracts were prepared by lysis in RIPA buffer (150 mM sodium chloride, 1.0% Triton X-100, 0.5% sodium deoxycholate, 0.1% SDS, 50 mM Tris, pH 8.0), supplemented with protease inhibitors (Roche). Protein concentration was determined by BioRad protein assay (BioRad) and 25 μg of proteins were separated on 13.5 % SDS-PAGE. After transfer to PVDF membrane and 45 min saturation in PBS/0.05% Tween 20/5% skim milk, membranes were incubated for 1 hour with anti-LC3IIb (D11, XP Rabbit monoclonal, Cell Signaling Technology) diluted as indicated by the manufacturer in PBS/0.05% Tween 20/0.5% skim milk, washed 3x10 min in PBS/0.05% Tween 20, incubated for 1 hour with the appropriate horseradish peroxidase-conjugated secondary antibody (BIO-RAD) and the signals detected with Chemiglow by means of a FluorChem SP system (AlphaInnotech). Anti-GAPDH (0411, Santa Cruz) was used as a control to determine equal protein loading. Signal intensities were quantified by means of ImageJ software (NIH, USA).

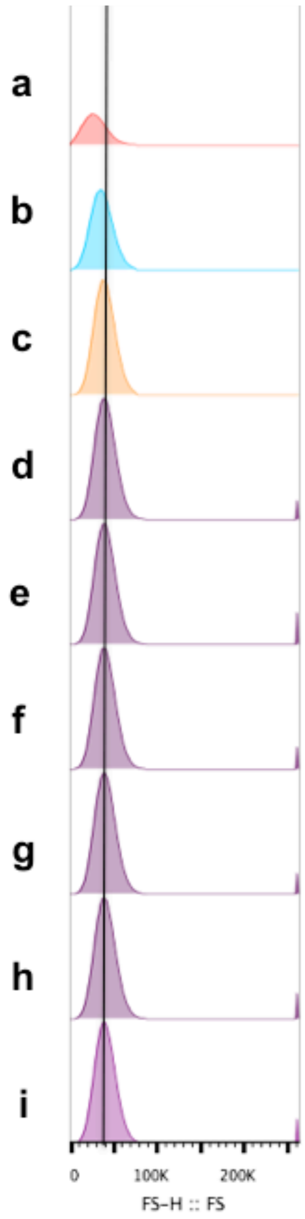

Supplementary Fig. 1. Dimensional analysis of ABLs. Panels A-I show Forward scatter (FS) distribution of beads of 0.6µm (**a**), 0.8µm (**b**) and 1 µm (**c**) and of ABL/PA (**d**), ABL/LBPA (**e**), ABL/AA (**f**), ABL/PI5P (**g**), ABL/PI3P (**h**) and ABL/S1P (**i**).

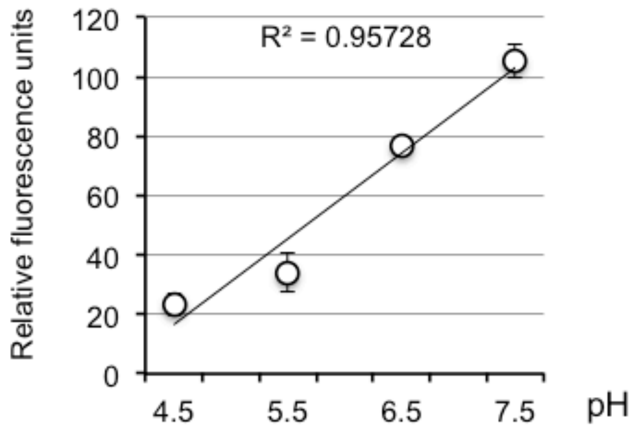

Supplementary Fig. 2. pH dependent fluorescence emission by BCG-NHS. BCG was labeled with 100 µg/ml of the pH sensitive dye N-hydroxysuccin-imidyl 5-(and 6-)-carboxyfluorescein (NHS-CF) in ice for 30 minutes. After washing, BCG-NHS was incubated in buffers calibrated at pH 4.5, 5.5, 6.5, 7.5 (intracellular pH calibration Buffer Kit, Molecular Probes). Results are expressed as mean ± standard deviation of relative fluorescence units performed in triplicate.

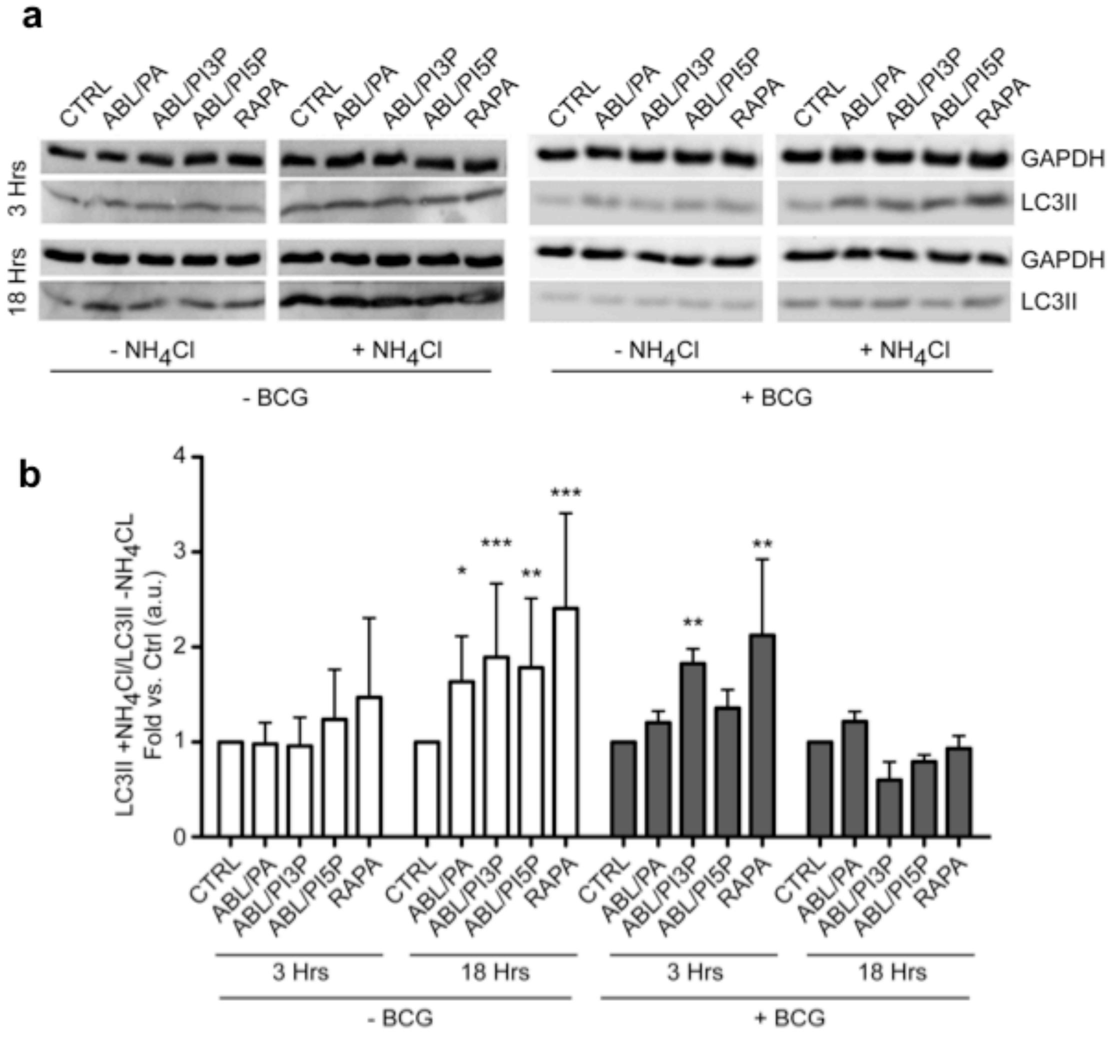

Supplementary Fig. 3. LC3II accumulation following stimulation with ABLs. (**a**) Western blotting detection of LC3II levels in BCG infected or control dTHP1 cells treated with indicated ABLs or rapamycin (RAPA) in the presence or absence of NH_4Cl, at 3 and 18 hours after stimulation. (**b**) LC3II levels were quantified by densitometric analysis and expressed as mean $\pm$ standard error of the ratio of LC3II in samples treated with ABLs in presence of NH_4Cl vs. the same samples in absence of NH_4Cl, both normalized for the loading control (GAPDH). Statistical analysis (n=5) has been performed by means of one-way ANOVA, and differences between samples with Bonferroni's post test. * $p<0.05$; ** $p<0.01$; *** $p<0.001$.

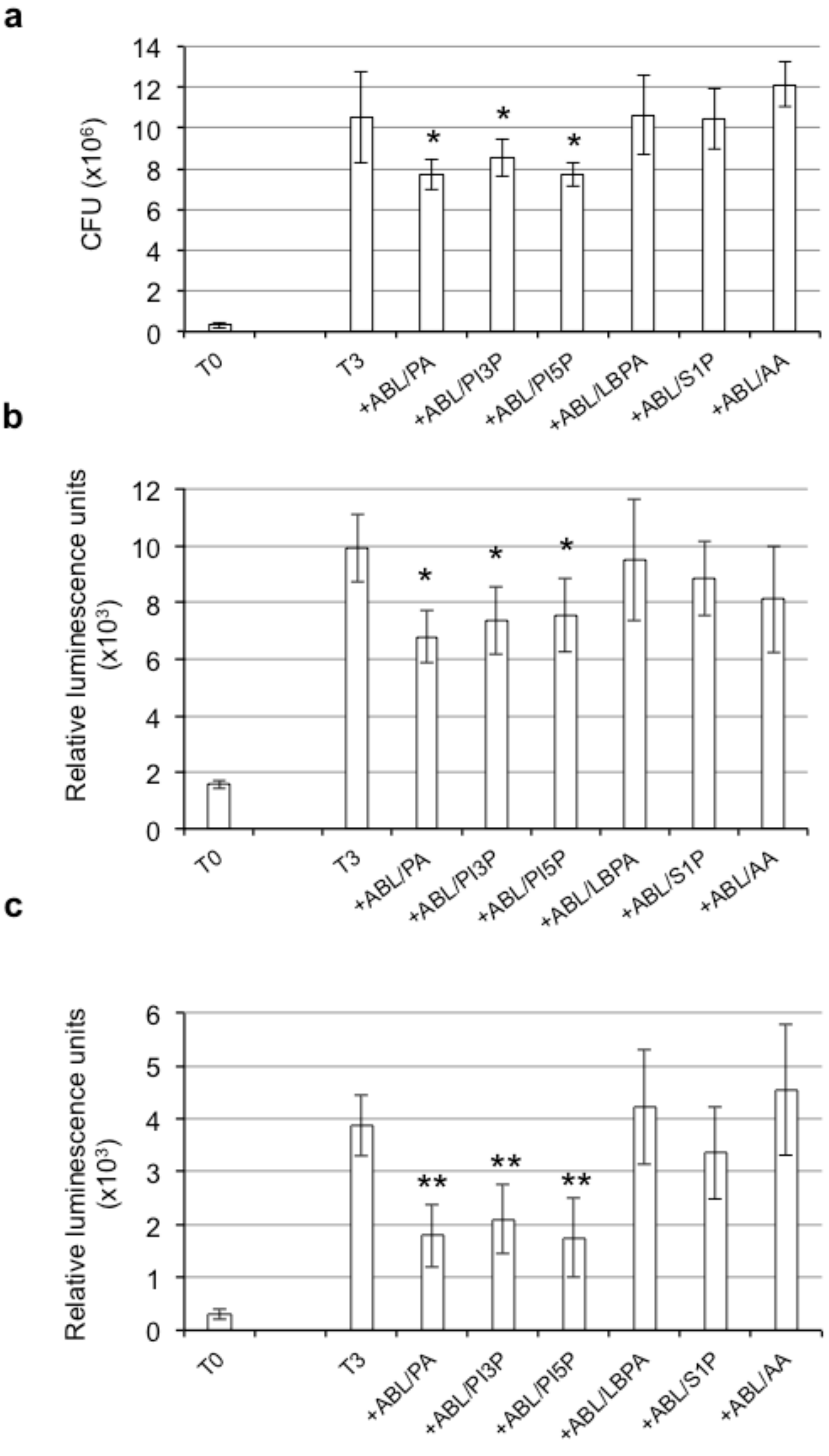

Supplementary Fig. 4. ABL carrying bioactive lipids promote intracellular mycobacterial killing. dTHP1 cells were infected with BCG (**a**) at the MOI of 5 or BCG-lux (**b**) at the MOI of 10 and then stimulated for 3 days with ABLs. Primary macrophages (**c**) were infected with BCG-lux at the MOI of 10 and then stimulated with ABLs for 3 days. Results are expressed as mean ± standard deviation of CFU values (**a**) or of relative luminescence units (**b,c**) performed in triplicate and are representative of two independent experiments. * $p < 0.05$ and ** $p<0.0001$ in comparison with non-stimulated control cells by one sided Student's *t* test.

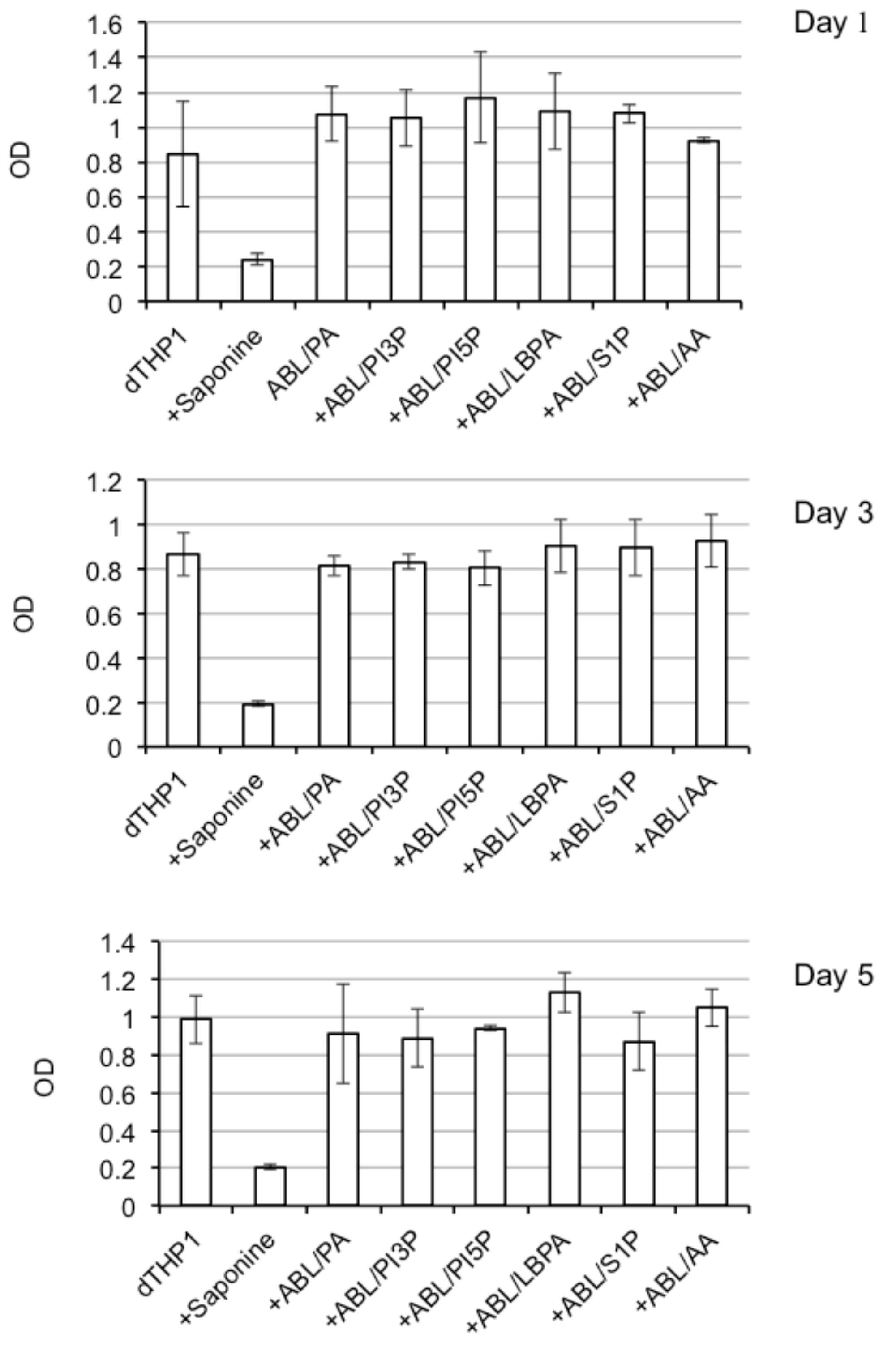

Supplementary Fig. 5. Evaluation of cell viability following stimulation with ABL. dTHP1 cells (10^6 cells/ml), were stimulated with the indicated ABLs for 1, 3 or 5 days and then cell viability was monitored by the MTT cell proliferation assay. The data are shown as mean ± standard deviation of the optical density values of triplicate cultures and are representative of 3 different independent experiments.

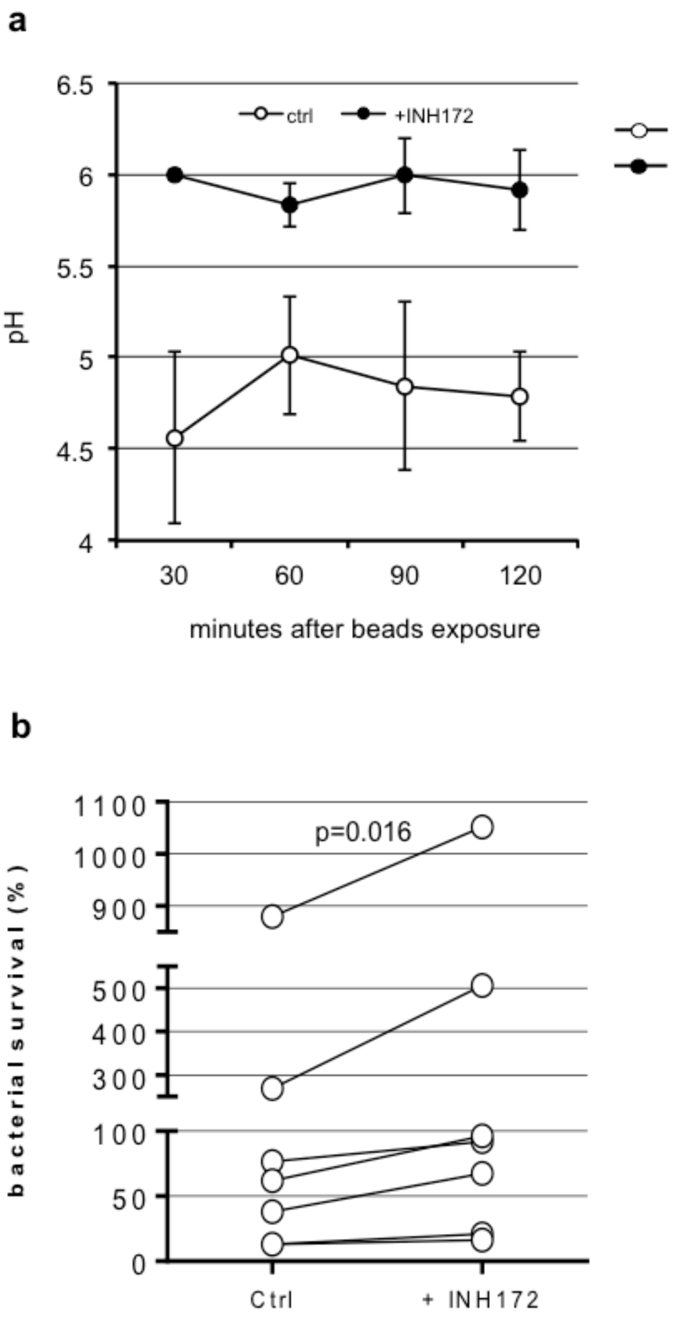

Supplementary Fig. 6. Pharmacological inhibition of CFTR inhibits phagosome acidification and promotes intracellular viability of *P. aeruginosa* in primary macrophages. Primary monocyte-derived macrophages (10^6 cells/ml), treated or not with INH-172 at a concentration of 10 µM, were exposed to latex beads (**a**) or infected with *P. aeruginosa* at the MOI of 30 (**b**). (**a**) pH was assessed by Lysosensor green DND 189 at the indicated times and results are shown as mean ± standard deviation of the values obtained from triplicate cultures. (**b**) Bacterial growth was assessed by CFU assay obtained immediately after infection (T0) and after 2 additional hours of incubation (T2). Bacterial survival is expressed as a percentage and calculated as the ratio between the CFU obtained at the time T2 and CFU obtained at the time T0, in the absence or presence of CFTR inhibitor INH-172. Statistical analysis was performed by using two sided Wilcoxon matched-pairs signed rank test and p value is indicated in the panels.

Supplementary Table 1. Demographic and clinical characteristics of cystic fibrosis (CF) patients and healthy controls (HC)

CF	Age	Gender	Genotype	Microbiology*	FEV1 (%)	HC	Age	Gender
**1**	32	M	F508del/2176insC	*P.a., S.a., B.c.*	67	**1**	30	M
**2**	29	M	F508del/F508del	*B.c.*	65	**2**	39	M
**3**	24	M	F508del/F508del	*S.a., A.f., S.ap.*	56	**3**	40	M
**4**	32	M	N1303K/M1V	*P.a.*	65	**4**	34	M
**5**	12	F	Q39X/microdeletion 4-11	*P.a.*	52	**5**	26	F
**6**	13	M	F508del/712-1G->T	*S.a.*	91	**6**	28	F
**7**	10	M	G542X/2183AA->G	*S.a.*	90	**7**	22	F
**8**	26	F	F508del/L1077P	*S.a., P.a., Sc.ap., S.m.*	61	**8**	27	F

** P.a.: Pseudomonas aeruginosa; S.a.: Staphylococcus aureus; B.c.: Burkholderia cepacia; A.f.: Aspergillus fumigatus; S.ap.: Scedosporium apiospermum; S.m.: Stenotrophomonas maltophilia*

Supplementary Table 2. Clinical features of pneumonia patients

**Patient**	**Sex**	**Age**	**Smoke** [A]	**Infectious agent**	**Provenance** [B]	**Type of LRTI** [C]	**Instilled/Recovered fluid [ml]**
#1	F	64	0	*K. pneumoniae*	2	2	120/45
#2	M	76	1	*K. oxytoca*	1	3	100/40
#3	F	55	1	*P. aeruginosa*	3	2	140/40
#4	F	81	0	*S. aureus*	3	2	90/20
#5	F	77	0	*A. baumannii*	3	1	100/40
#5	F	77	0	*E. coli*	3	1	100/40
#6	F	76	1	*P. aeruginosa*	1	1	100/40

A 0 = no smoker; 1 = ex smoker

B 1 = house; 2 = Emergency department; 3 = ICU/Medical Units

C LRTI = lower respiratory tract infection; 1 = CAP (community acquired pneumonia); 2 = HAP (hospital acquired pneumonia); 3 = AEBX (acute exacerbation of bronchiectasis)

Patient #5 is a coinfection by *A. baumannii* and *E. coli*
